# Supplementary material for: Upcycling of polyamides through chemical hydrolysis and engineered Pseudomonas putida
Source: Nat Microbiol. 2025 Feb 10;10(3):667–80. doi: 10.1038/s41564-025-01929-5 (PMC11879879; doi:10.1038/s41564-025-01929-5)
Supplement: Supplementary file 1 — Reporting Summary [file 41564_2025_1929_MOESM1_ESM.pdf]

nature portfolio

Corresponding author(s): Nick Wierckx

Last updated by author(s): Dec 4, 2024

Reporting Summary

Nature Portfolio wishes to improve the reproducibility of the work that we publish. This form provides structure for consistency and transparency in reporting. For further information on Nature Portfolio policies, see our Editorial Policies and the Editorial Policy Checklist.

Statistics

For all statistical analyses, confirm that the following items are present in the figure legend, table legend, main text, or Methods section.

n/a	Confirmed	
☐	☒	The exact sample size (*n*) for each experimental group/condition, given as a discrete number and unit of measurement
☐	☒	A statement on whether measurements were taken from distinct samples or whether the same sample was measured repeatedly
☐	☒	The statistical test(s) used AND whether they are one- or two-sided<br>*Only common tests should be described solely by name; describe more complex techniques in the Methods section.*
☒	☐	A description of all covariates tested
☒	☐	A description of any assumptions or corrections, such as tests of normality and adjustment for multiple comparisons
☐	☒	A full description of the statistical parameters including central tendency (e.g. means) or other basic estimates (e.g. regression coefficient) AND variation (e.g. standard deviation) or associated estimates of uncertainty (e.g. confidence intervals)
☐	☒	For null hypothesis testing, the test statistic (e.g. *F*, *t*, *r*) with confidence intervals, effect sizes, degrees of freedom and *P* value noted<br>*Give P values as exact values whenever suitable.*
☒	☐	For Bayesian analysis, information on the choice of priors and Markov chain Monte Carlo settings
☒	☐	For hierarchical and complex designs, identification of the appropriate level for tests and full reporting of outcomes
☒	☐	Estimates of effect sizes (e.g. Cohen's *d*, Pearson's *r*), indicating how they were calculated

Our web collection on statistics for biologists contains articles on many of the points above.

Software and code

Policy information about availability of computer code

Data collection	No software other than the respective device's onboard programmes was used to collect the data.
Data analysis	Analysis of WGS and RNA-Seq was performed using CLC genomics workbench v.20 (Qiagen, Germany). Promoters were predicted using SAPPHIRE. Predictions of protein domains were performed with InterPro. Operons were predicted using the Operon Mapper. Protein structures were predicted using ColabFold 1.5.5. DNA and protein sequences were aligned to the nucleotide collection (nr/nt) of the NCBI database using BLASTn and BLASTp. Gene annotations were performed based on the Pseudomonas genome database that can be accessed via https://pseudomonas.com/. Data on bacterial growth and enzyme activity were analysed using MS Excel2016 and GraphPad Prism 8.1.2.332 and Prism 10.3.1. HPLC data was processed with Agilent OpenLab Data Analysis - Build 2.204.0.661 and analysed using MS Excel2016 andGraphPad Prism 8.1.2.332 and Prism 10.3.1. Molecules and protein complexes were visualized using ChemDraw 18.0.0.231 (PerkinElmer, Shelton, CT, USA).

For manuscripts utilizing custom algorithms or software that are central to the research but not yet described in published literature, software must be made available to editors and reviewers. We strongly encourage code deposition in a community repository (e.g. GitHub). See the Nature Portfolio guidelines for submitting code & software for further information.

Data

Policy information about availability of data

All manuscripts must include a data availability statement. This statement should provide the following information, where applicable:

- Accession codes, unique identifiers, or web links for publicly available datasets
- A description of any restrictions on data availability
- For clinical datasets or third party data, please ensure that the statement adheres to our policy

All relevant data is presented in the manuscript or the supplementary information. Sequencing data are stored in the NCBI Sequence Read Archive under BioProject PRJNA1023861; transcriptomic data were deposited at GEO of NCBI under accession number GSE244960 and has been made public on Nov 20, 2024:All relevant data is presented in the manuscript, the Extended Data, the supplementary information and the respective source files.. Spreadsheets with the source data of the depicted diagrams is provided with the manuscript.

Research involving human participants, their data, or biological material

Policy information about studies with human participants or human data. See also policy information about sex, gender (identity/presentation), and sexual orientation and race, ethnicity and racism.

Reporting on sex and gender	N/A
Reporting on race, ethnicity, or other socially relevant groupings	N/A
Population characteristics	N/A
Recruitment	N/A
Ethics oversight	N/A

Note that full information on the approval of the study protocol must also be provided in the manuscript.

Field-specific reporting

Please select the one below that is the best fit for your research. If you are not sure, read the appropriate sections before making your selection.

☒ Life sciences ☐ Behavioural & social sciences ☐ Ecological, evolutionary & environmental sciences

For a reference copy of the document with all sections, see nature.com/documents/nr-reporting-summary-flat.pdf

Life sciences study design

All studies must disclose on these points even when the disclosure is negative.

Sample size	N=3 was chosen as a suitable sample size due to practical constraints. This is a commonly used sample size for enzymatic reactions and biotransformations, and comperative studies on bacterial growth, and proved to be sufficient to yield reliable results (doi:10.1186/ s12934-024-02310- doi:10.1016/j.ymben.2022.12.008 doi:10.3389/fmicb.2020.00382.) This sample size was determined to be adequate based on experimental consistency and small standard deviations of n=3 in the shown experiments, hence it is unlikely that additional information would be gained from further replicates.
Data exclusions	No data was excluded
Replication	Growth data was collected using three independent cultures of each strain/each condition. Enzymatic reactions were likewise conducted in triplicates. All replicates were succesful.
Randomization	Experimental design was not subjected to randomization due to practical constraints involving large data spaces and the hypothesis-driven nature of the research requiring selection of specific targets from e.g ALE experiments. Neither animals nor human participants were involved in this study.
Blinding	The researchers conducting the experiments were not blinded as information strains/enzymes and substrates was necessary to set up cultures, reactions and specific analytics used and to then link the data back to the strains/enzymes and substrates. No other experiments other than those described were conducted in the study.

Reporting for specific materials, systems and methods

We require information from authors about some types of materials, experimental systems and methods used in many studies. Here, indicate whether each material, system or method listed is relevant to your study. If you are not sure if a list item applies to your research, read the appropriate section before selecting a response.

Materials & experimental systems

n/a	Involved in the study
☒	☐ Antibodies
☒	☐ Eukaryotic cell lines
☒	☐ Palaeontology and archaeology
☒	☐ Animals and other organisms
☒	☐ Clinical data
☒	☐ Dual use research of concern
☒	☐ Plants

Methods

n/a	Involved in the study
☒	☐ ChIP-seq
☒	☐ Flow cytometry
☒	☐ MRI-based neuroimaging

Plants

Seed stocks	N/A
Novel plant genotypes	N/A
Authentication	N/A
